# Factors related to the return to work of head and neck cancer patients diagnosed between 2004–2011 in Belgium: a multivariate Fine-Gray regression model analysis

**DOI:** 10.1186/s13690-024-01373-7

**Published:** 2024-09-12

**Authors:** Maxim Van den Broecke, Sarah de Jong, Katrien Vanthomme, Régine Kiasuwa Mbengi, Christophe Vanroelen

**Affiliations:** 1 Belgian Cancer Center, Belgian National Institute of Public Health (Sciensano), Brussels, Belgium; 2 Brussels Institute for Social and Population Studies, Vrije Universiteit Brussel, Brussels, Belgium; 3 METICES Research Group, Faculty of Sociology, Université Libre de Bruxelles, Brussels, Belgium; 4 Department of Public Health and Primary Care, Ghent University, Ghent, Belgium

**Keywords:** Head and neck cancer, Employment status, Return to work, Fine-gray analysis

## Abstract

**Background:**

This study aims to identify the key factors that underlie the return to work (RTW) of head and neck cancer (HNC) patients in Belgium.

**Methods:**

We used data from the EMPCAN database linking data from the Belgian Cancer Registry and the Crossroads Bank for Social Security. We selected HNC patients aged 18–60 at diagnosis who became inactive on the labour market during the follow-up time observed (*n* = 398). Fine-Gray regression models were used to examine associations between clinical, socio-demographical and work-related factors and RTW over a follow-up of almost 8 years (2004–2011).

**Results:**

The overall RTW was 21.6%. Stage IV at diagnosis and the use of chemoradiation were associated with a decreased RTW probability but this effect was attenuated by age-adjusted analyses. Multivariate analysis shows that the probability of RTW decreases with age and depends on the household composition. Patients who live alone (SHR 2.2, 95% CI 1.0 – 4.5) and patients who live with another adult and child(ren) (SHR 2.1, 95% CI 1.1 – 4.0) are more likely to RTW than patients who live with another adult without children.

**Conclusions:**

The cumulative incidence of RTW in HNC patients is associated with age and household composition but not with treatment modalities or stage. In future research, this model could be applied to larger cancer patient groups for more accurate estimations. These insights are of importance to better support patients and for informing tailored policy measures which should take into account the sociodemographic profile of HNC patients to tackle societal and health-related inequities and burden of work inactivity.

**Supplementary Information:**

The online version contains supplementary material available at 10.1186/s13690-024-01373-7.

**Table:** 

Text box 1. Contributions to the literature
• Survival analysis methods have been used to investigate RTW but the current literature does not take into account competing risks or events such as retirement or death although this enables more accurate estimates and understanding of RTW among cancer patients.
• The extent to which different treatment modalities, which are assumed to impact physical functioning in different ways, affect RTW outcomes for HNC head and neck cancer patients remains unclear.
• This study emphasizes the need to develop tailored RTW interventions or policies that take into account sociodemographic differences to support and guide the RTW of head and neck cancer patients.

## Introduction

Worldwide, approximately 900 000 cases of head and neck cancers (HNCs) are diagnosed annually [1]. In 2020, 2 531 Belgians were diagnosed with a HNC [2]. Belgian retrospective cohort studies from 2004–2008 and 2017–2021 show that 5-year survival rates for HNC patients slightly increased (men: 50% to 54.9%, women: 57.0% to 60.2%) [3–6], which can be attributed to enhanced treatments and growing human papillomavirus (HPV) related HNC cases, known for better outcomes [3]. This trend coincides with rising HNC incidences in women and younger men, linked to more HPV infections and increased tobacco use among women [7].

Similarly to overall cancer survivors, HNC survivors face numerous adverse effects on both their health and their social life as a result of the disease and its treatment [8]. These include general effects experienced by cancer patients, such as fatigue and pain [9], as well as effects specific to HNC such as permanent functional and sensory changes after radiation therapy (RT) [10] generally leading to the need for life-long care and support to maintain basic life functions and quality of life [11]. Likewise, surgery has been associated with a significantly reduced health-related quality of life, with speech and day-to-day activities being the most affected dimensions [12]. Chemoradiation therapy (CRT) – i.e., chemotherapy (CT) combined with RT – has been significantly associated with pharyngeal-laryngeal toxicity compared to RT as a unimodal therapy [13].

The treatment of HNCs may also negatively affect mental health, social functioning, employment and family interactions [8]. Work inability has important financial implications at the societal level (e.g., high cost due to lost work days and social benefits) [14] but also for the well-being of individuals and their household as a result of psychological distress in both patients and their families [12, 13]. Epidemiological studies report that HNC patients in particular are more at risk to become or remain unemployed in comparison to overall cancer survivors [15–17]. Possible explanations for this relate to long-term effects that are unique to HNC treatment such as difficulty in speaking and visible effects of surgery [12]. With HNC incidences increasing among working-aged individuals [18], increased attention should be given to rehabilitation and survivorship strategies that support reintegration into the workforce.

A systematic review published in 2022 on the return to work (RTW) of HNC patients [17] investigated possible determinants for RTW such as patient characteristics, clinical factors, work-related factors, etc. Important variations in reported RTW rates (from 32 to 90% [17]) were identified and were (partly) attributed to the heterogeneity between studies in terms of, among others, study design and definitions of work-related outcomes. For age and sex, the majority of studies found older age to be negatively associated with RTW and sex to have no association with RTW [17]. Out of the thirteen studies examining the association between RTW and treatment modalities, three studies [19–21] found a significant negative association between RTW and multimodal therapy compared to unimodal treatment. One survey-based study showed that HNC patients who received more than one type of therapy were 5.9 times more likely to report work-related disability [21]. For both CT and RT as unimodal therapies, no studies found an association with RTW. Two out of thirteen studies [22, 23] found surgery to be significantly and negatively associated with RTW. Finally, three out of thirteen included studies found that advanced tumour stage was negatively associated with RTW in HNC survivors [19, 20, 24].

Compared to RTW literature related to other cancer sites, studies focussing on RTW in HNC patients are sparse [25]. Also, the effects of therapeutic modalities on the RTW are less often addressed nor reported in studies focussing on HNC patients compared to other cancers [25]. Moreover few studies have managed to investigate RTW in HNC patients using register data at a national level. Therefore, this study makes use of population-level data on HNC patients to investigate RTW probabilities and its determinants in Belgium. More specifically, this study examined the RTW of HNC patients diagnosed between January 2004 and December 2011, aged 18–60 at the moment of diagnosis and who experienced a period of work inactivity after the diagnosis. The data originated from the EMPCAN database which describes clinical, socio-demographical, and quarterly work- and social security related information on cancer patients in Belgium. Work inactivity is presently defined as inactivity on the labour market for at least 28 consecutive days on the last day of the quarter.

The data originates from the EMPCAN database for which data from the Belgian Cancer Registry were linked with data from the Belgian Crossroads Bank for Social Security (CBSS), at the individual level. More specifically, this study investigates the association between clinical, socio-demographical and work-related factors and the RTW of HNC patients, during a maximum follow-up of 7 years and 9 months.

## Methods

### HNC patients in the empcan database

The data used for this study were derived from the EMPCAN database [26] which includes patients of working age (aged 16–64), diagnosed between January 1st 2004 and December 31st 2011, with cancers of the breast, colorectal, head and neck, corpus uterus, prostate, testis and lung who were active on the labour market in the quarter prior to their data of diagnosis. The Belgian Cancer Registry provided the EMPCAN database with information on the month of incidence, cancer type, stage of disease at diagnosis and treatment modalities received during the first year after incidence. The Belgian Crossroads Bank for Social Security (CBSS) provided quarterly information on the labour market position starting from the quarter of diagnosis up to December 31st 2012. Based on information from the CBSS, all workers who were registered as disabled, unemployed or on sick leave in the three quarters prior to the month of incidence were excluded. The result was a cohort of 41 678 workers with cancer. The methods that were used to link the databases at the individual level and the detailed list of variables are described in a previous article [26].

**Fig. 1:**
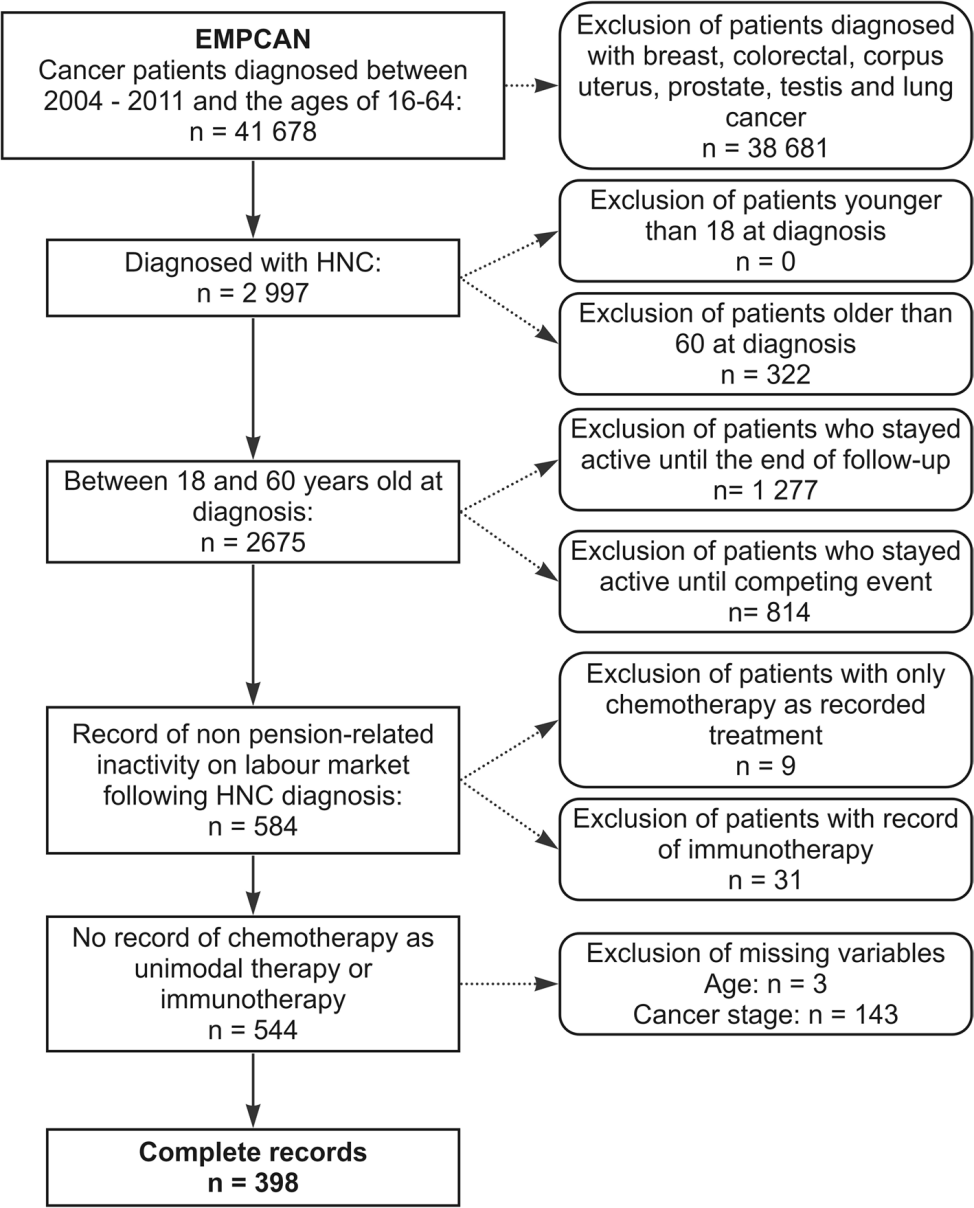
Flowchart for selection of individuals in the EMPCAN database with record of labour market inactivity following a head and neck cancer diagnosis, between 2004 and 2011 in Belgium

The EMPCAN database was further restricted in a step-wise manner to allow for the analyses envisaged within this study (Fig. 1). Patients diagnosed with a cancer other than HNC (*n* = 38 681) and patients older than 60 at the moment of diagnosis (*n* = 322), who would reach formal (early) retirement age (from 63 years for women and 65 years for men in 2004 to 65 years for both in 2012 with the possibility of early retirement 5 years earlier based on years of labour market activity) during follow-up, were excluded. The resulting database of 2 675 patients was further restricted to exclude patients who remained active on the labour market until the end of follow-up or until death or retirement (*n* = 2 091). Also, patients who received treatment modalities that do not align with guidelines for HNC developed by the Belgian College of Oncology [27, 28] were excluded. This relates to immunotherapy (*n* = 31), and CT as unimodal therapy (*n* = 9) which are indicative of differing or more complex pathologies or multimorbidity. Finally, patients for whom missing values are present in the variables of interest (see *3.2. measures*) were excluded (*n* = 143). With the latter, a relatively large proportion of the sample (26.3%) was excluded, marking a limitation of the present study. Finally, a database comprising 398 HNC patients was established.

### Measures

#### Return to work, retirement and death

The EMPCAN database contains quarterly information on the position on the labour market and on the type of social benefits granted. These included: career pause, early pension, disability insurance, invalidity, unemployment, exempt from job search, compensation for a handicap, being a child entitled to benefits, work accident and illness, social integration income and ‘other’. Moreover, a dichotomous variable that tells if a patient was alive or dead at the time of the patient’s last record in the database is available in the EMPCAN database.

Based on this information, three events can be distinguished:

1. RTW: the first quarterly record of being active (either part-time or full-time) on the labour market following a first period of inactivity after a HNC diagnosis was used to define RTW in the EMPCAN database. In Belgium, sickness absence benefits of employees are covered by the employer for the first 28 consecutive days. After that, they enter into primary work inactivity for a maximum of 11 months (paid by social benefits). After one year of consecutive sickness absence, they enter into invalidity. Due to the quarterly nature of the CBSS data on labour market positions, the current study population can be further specified as HNC patients who, after receiving their diagnosis, were inactive for at least 28 days at the last day of the quarter following the diagnosis. The first record of inactivity was allowed to take place at any time after the diagnosis.
2. Death: recorded by a distinct dichotomous variable derived from the CBSS.
3. Retirement: constructed based on above-mentioned information on the types of social benefits (as they cannot be combined with formal retirement, except the compensation for handicap). Patients older than 50 years who have not died nor returned to work during the follow-up, and with continuous records of inactivity on the labour market without receiving the above-listed social benefits (excl. handicap compensation, early retirement or bridge pension) were regarded as entering retirement [29].

In this study, RTW is the event of interest while retirement and death are competing events. The analyses are based on the occurrence of these events and the corresponding time-to-event. Time-to-event for RTW was determined by the quarter at which inactivity on the labour market was first recorded and the quarter at which patients first became active again. Time-to-event for retirement and death was similarly defined. Patients who experienced no event after a measure of inactivity on the labour market were censored at the end of their respective follow-up times, i.e. they remained inactive.

### Explanatory variables

For this study, three groups of explanatory variables were identified in the EMPCAN database: socio-demographic [age (at diagnosis), gender, household composition], work-related (occupational class), and disease-related (combined stage and therapeutic modalities) explanatory variables. At present, the majority of studies on factors related to the RTW of HNC patients find no association between sex or family circumstances (incl. marital status and household composition) and RTW [17], with notable exceptions [30–32]. In the present study, household composition categorizes an individual as either living alone, living with a partner, living with child(ren) and a partner and being a single parent. Occupational class has been found to be associated with RTW in HNC patients with white collar workers being more likely to return to work, although these results are not confirmed by all eligible studies [17]. The EMPCAN database distinguishes between blue collar workers (essentially performing manual work), white collar workers (seen as having intellectual jobs) and civil servants. The ‘other’ outcome level of the occupational class variable refers to people living in Belgium with an occupation that was not classified as blue collar, white collar or civil servant (e.g., self-employed, helping partner or housewives).

At present, studies that examine factors related to the RTW of HNC patients offer no consensus on the extent to which clinical factors such as tumour site, stage and treatment are associated with RTW [17]. Only a minority of studies find that advanced tumour stage or multimodality treatment is negatively associated with the RTW of HNC patients. For this study, therapeutic modalities were initially recorded as dichotomous variables that represent whether patients have received RT or CT in the 12 months after their diagnosis. Reliable records for surgery were unavailable at the time when the EMPCAN database was developed [4]. Surgery was considered as unimodal therapy and deducted based on those patients for whom no other treatment was recorded. This limitation means that for patients who received chemotherapy or radiation therapy, the current study is unable to provide more detail on whether surgery took place or not. Therapeutic modality was finally recoded into three categories that reflect the clinical guidelines from the Belgian College of Oncology [27, 28]: surgery as unimodal therapy, radiation therapy and chemoradiation therapy. For this study, combined stage refers to a merged stage based on both clinical and pathological stages of cancer.

### Analysis

Associations between RTW and explanatory variables were assessed by using competing risks regression analyses based on the Fine-Gray proportional hazards model [33]. In standard survival analysis, since not all patients experience the event of interest by the end of the study period, their unknown survival times are censored to allow for valid inferences [30]. However, competing risks can occur and represent the risk of an event whose occurrence precludes the event of interest [33]. Taking this into account, the Fine-Gray model allows for a more accurate examination of which covariates affect the probability of an event (RTW) occurring over time [34].

First, assuming the presence of competing risks, the cumulative incidence (CI) of RTW over the total follow-up period was assessed and reported as well as the CI after one, three and five years of work inactivity. Gray’s test for subdistribution hazards was used to compare cumulative incidence functions (CIF) for the entire follow-up time for different levels of each explanatory variable [35].

Next, uni- and multivariate analyses were conducted to look for the effects of the explanatory variables on RTW using subdistribution hazards ratios (SHRs) and 95% confidence intervals. Ahead of this, multicollinearity assumptions between the explanatory variables were checked using the Chi-Square test of independence (supporting aggregated data is available in ‘Extra file 1’). As age was found to be highly correlated with all variables except for cancer stage, age-adjusted SHRs were calculated as well as fully adjusted SHRs in a multivariate competing risk regression model. Taking into account the low number of individuals in the youngest two age categories (18–25 years old and 26–35 years old), we performed a sensitivity analysis in which these two categories were combined. To further investigate the effect of age on our analyses, stratified multivariate analyses were performed for patients aged 50 years or less and patients older than 50 separately.

The analyses were performed in RStudio (version 2024.4.2.764) using the *tidycmprsk* package.

## Results

In our study, patients experienced varying lengths of follow-up, with times ranging from 1 to 31 quarters. The average (mean) follow-up time was 7 quarters, while the median follow-up period was 4 quarters, indicating a wide distribution in the duration of observation among participants. During the follow-up period, 86 patients (21.6%) successfully returned to work. Additionally, 26 patients (6.5%) retired, 126 patients (31.7%) passed away, and 160 patients (40.2%) were censored, meaning they remained inactive until the end of the follow-up period.

**Fig. 2:**
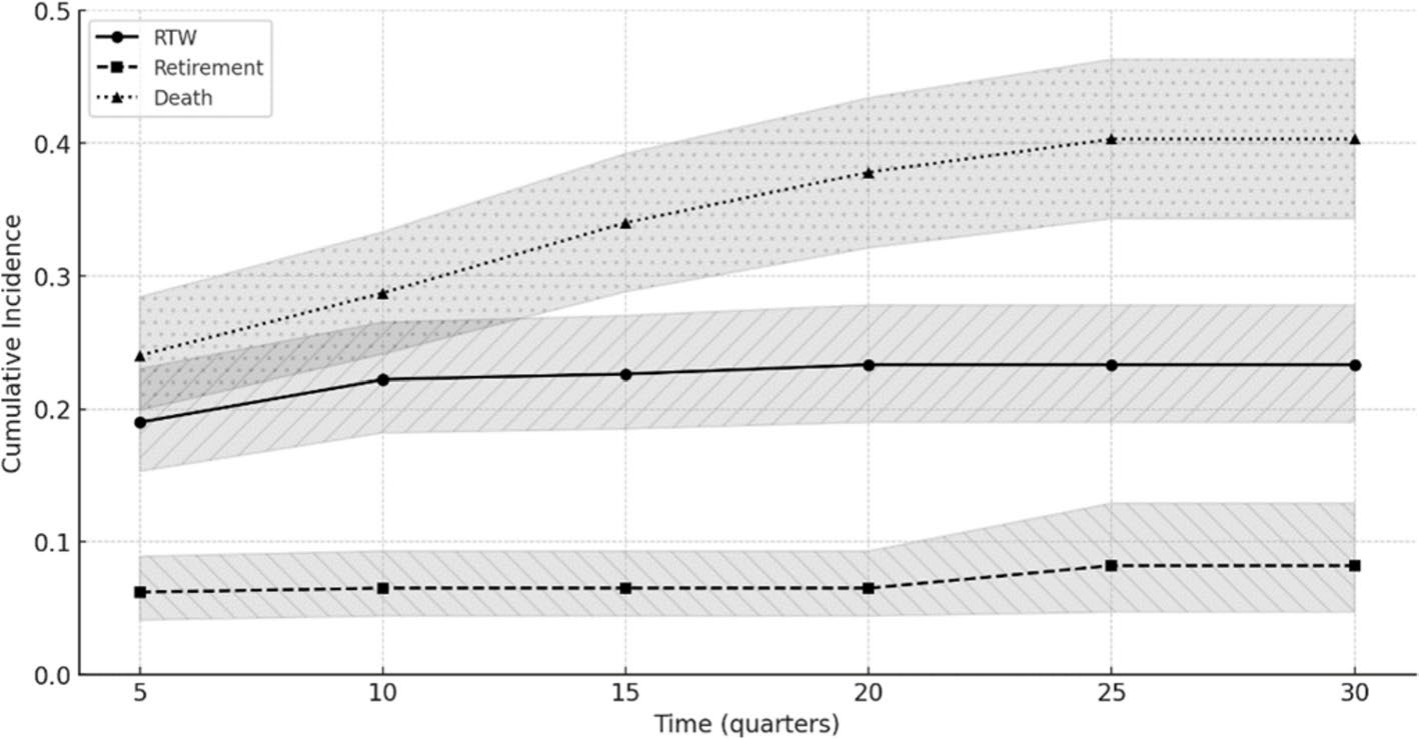
Cumulative incidence of return to work, retirement and death during 30 quarters of follow-up after a head and neck cancer diagnosis in Belgium, between 2004 and 2011

Patient characteristics are shown in Table 1, as well as the CI of RTW at one, three and five year after diagnosis. The study population predominantly consisted of individuals aged 51–60 years (66.8%), with a significant majority being men (81.2%). Most patients were either co-habiting (37.9%) or living with a child (34.7%). Over half were classified as 'Other' in occupational class (56.5%) while, respectively, 19.4% and 10.1% were blue and white collar workers. Regarding cancer stage, the largest group was diagnosed with Stage IV (39.7%), and the most common therapeutic modalities were chemoradiation therapy (38.7%) and radiation therapy (36.4%). Figure 2 shows that RTW rapidly increases up to quarter 10 (2.5 years) and then reaches a plateau at quarter 12 (3 years).

**Table 1:** Patient characteristics and cumulative incidence of return to work after one, three and five years of work inactivity following a head and neck cancer diagnosis between 2004 and 2011 in Belgium

			CI (%) and 95% Confidence Intervals for the RTW after 1, 3 and 5 years of work inactivity			
	N (N_Excl_)	% (%_Excl_)	Year 1	Year 3	Year 5	P*
Sociodemographic explanatory variables						
Age						< 0.001
18–25	6 (9)	1.5 (0.6)	67 (14–92)	-	-	
26–35	12 (51)	3.0 (3.6)	61 (26–84)	-	-	
36–50	114 (472)	28.6 (32.9)	26 (18–34)	31 (23–40)	32 (24–44)	
51–60	266 (902)	66.8 (62.9)	19 (9–17)	14 (10–19)	14 (10–19)	
Sex						0.03
Men	323 (1165)	81.2 (81.2)	17 (13–22)	20 (15–25)	21 (16–26)	
Women	75 (269)	18.8 (18.8)	25 (15–35)	35 (23–47)	35 (23–47)	
Household composition						< 0.001
Co-habitant	151 (439)	37.9 (30.6)	12 (7–17)	12 (7.7–18)	12 (7.7–18)	
Co-habitant with child	138 (577)	34.7 (40.2)	29 (21–36)	34 (26–42)	34 (26–42)	
Single	71 (292)	17.8 (20.4)	19 (11–29)	26 (16–37)	26 (16–37)	
Single with child	29 (102)	7.3 (7.1)	10 (2.6–25)	18 (6.4–35)	18 (6.4–35)	
Other	9 (24)	2.3 (1.7)	11 (0.47–41)	11 (0.47–41)	11 (0.47–41)	
Work-related explanatory variable						
Occupational class						0.071
blue collar	77 (350)	19.4 (24.41)	13 (6.8–22)	17 (9.2–27)	19 (11–30)	
white collar	40 (253)	10.1 (17.64)	20 (9.5–34)	27 (14–42)	27 (14–42)	
civil servant	56 (207)	14.1 (14.44)	9.1 (3.3–18)	11 (4.5–21)	11 (4.5–21)	
Other	225 (624)	56.5 (43.51)	23 (17–28)	27 (21–33)	27 (21–33)	
Disease-related explanatory variables						
Cancer stage						0.058
I	113 (383)	28.4 (26.7)	25 (18–34)	32 (23–42)	32 (23–42)	
II	64 (226)	16.1 (15.8)	16 (8.2–26)	20 (11–31)	25 (13–39)	
III	67 (226)	16.8 (15.8)	19 (11–30)	23 (14–35)	23 (14–35)	
IV	154 (599)	39.7 (41.8)	14 (9.4–21)	17 (11–23)	17 (11–23)	
Therapeutic modality						0.071
Surgery	99 (309)	24.9 (21.6)	15 (9.5–21)	17 (11–23)	17 (11–23)	
Radiation therapy	145 (431)	36.4 (30.1)	19 (13–26)	23 (17–31)	25 (18–33)	
Chemoradiation therapy	154 (694)	38.7 (48.4)	25 (17–34)	30 (21–40)	30 (21–40)	

*N* Number of patients. N_Excl_ = Number of patients who were excluded. %_Excl_ = Proportion of excluded patients in each category. *CI* Cumulative Incidence. *Gray’s test; *p* < 0.05 = Significantly different CI by level of explanatory variables

The Gray’s test showed that for the different age groups, sexes and household compositions, the CI’s are significantly different over the total follow-up time (see Table 1). Patients in younger age groups (18–25 and 26–35) and women (16% of whom are aged < 36 compared to 2.8% of men) present higher RTW rates compared to older age groups and men. In the youngest age groups (< 36 years), all patients (*n* = 18) returned to work within the 3 years after diagnosis. Patients who live with a partner and child(ren) returned to work more often than patients who live in differently composed households. Differences between occupational classes, cancer stages or therapeutic modalities were not significant.

Table 1 shows that for most explanatory variables, the CI’s increased during the first three years following the first record of work inactivity, although the significance of these increases was not assessed. Between years three and five, no increase in the CI’s is observed with the exception of patients diagnosed with stage ll and patients having received radiation therapy (yet modestly, with respectively five and two percentiles).

Table 1 also reports the characteristics of the individuals who were excluded (N_Excl_, %_Excl_) because they of not having experienced inactivity (i.e. more than 3 months). After applying the same exclusion criteria for treatment modalities and missing values, the distributions of individuals across the categories of each explanatory variable are similar to the included HNC patients. Chi-square test were performed to identify significantly differing distributions between individuals who were included and excluded, across the outcome levels from each explanatory variable. Significant differences were found only for occupational class (χ^2^ = 26.074, *p* < 0.001) and therapeutic modality (χ^2^ = 11.912, *p* = 0.003).

Associations between the explanatory variables and RTW were examined with univariate competing risk models (Table 2). The analyses showed that, compared to patients aged 36–50, those aged 18–25 and 26–35 have a significantly higher probability of becoming active again on the labour market [SHR 4.3 (95% CI 3.0 – 6.3) and SHR 3.0 (95% CI 1.7 – 5.2) respectively, *p* < 0.001]. Being female was also associated with a higher RTW probability [SHR 1.6 (95% CI 1.1 – 2.6), *p* < 0.001] compared to being male. Cohabiting adults with child(ren) [SHR 3.0 (95% CI 1.8 – 5.1), *p* < 0.001] and single HNC patients without child(ren) [SHR 2.3 (95% CI 1.2 – 4.3), *p* < 0.05] have a significantly higher probability of RTW compared to those cohabiting without child(ren). No significant association between RTW and occupational class was found.

**Table 2:** Return to work after work inactivity in head and neck cancer patients diagnosed between 2004 and 2011 in Belgium. Univariate, age-adjusted and fully adjusted (Subdistribution) Hazard Ratios

	Univariate SHR (95% CI) [*p*-value]	Age-adjusted SHR (95% CI) [*p*-value]	Mutually adjusted SHR (95% CI) [*p*-value]
Sociodemographic explanatory variables			
** Age**			
36–50	*Ref.*	*Ref.*	*Ref.*
18–25	4.3 (3.0 – 6.3) [< 0.001]		3.3 (1.9 – 5.8) [*p* < 0.001]
26–35	3.0 (1.3 – 5.2) [< 0.001]		2.7 (1.3 – 5.5) [0.008]
51–60	0.4 (0.3 – 0.6) [< 0.001]		0.5 (0.3 – 0.8) [0.005]
** Sex**			
Man	*Ref.*	*Ref.*	*Ref.*
Woman	1.6 (1.1 – 2.6)* [0.031]	1.08 (0.7 – 1.7) [0.78]	1.0 (0.6 – 1.7) [0.86]
** Household composition**			
Cohabitant	*Ref.*	*Ref.*	*Ref.*
Cohabitant with child	3.0 (1.8 – 5.1) [< 0.001]	1.9 (1.1 – 3.6) [0.033]	2.1 (1.1 – 4.0) [0.018]
Single	2.3 (1.2 – 4.3) [0.011]	1.8 (0.9 – 3.7) [0.1]	2.2 (1.1 – 4.5) [0.034]
Single with child	1.4 (0.6 – 3.8) [0.45]	1.0 (0.42 – 2.3) [0.96]	1.1 (0.4 – 2.6) [0.9]
Other	1.0 (1.1 – 7.2) [0.97]	0.8 (0.1 – 6.7) [0.86]	1.0 (0.1 – 7.9) [0.97]
Work-related explanatory variable			
** Occupational class**			
Blue collar	*Ref.*	*Ref.*	*Ref.*
White collar	1.5 (0.7 – 3.4) [0.3]	1.1 (0.5 – 2.4) [0.8]	1.2 (0.6 – 2.7) [0.59]
Civil servant	0.6 (0.2 – 1.6) [0.32]	0.7 (0.3 – 1.6) [0.37]	0.7 (0.3 – 1.7) [0.41]
Other	1.5 (0.9 – 2.7) [0.15]	1.6 (0.9 – 2.8) [0.12]	1.8 (1.0 – 3.3) [0.052]
Disease-related explanatory variables			
** Cancer stage**			
I	*Ref.*	*Ref.*	*Ref.*
II	0.7 (0.4 – 1.2) [0.18]	0.6 (0.4 – 1.1) [0.1]	0.6 (0.4 – 1.1) [0.12]
III	0.7 (0.4 – 1.3) [0.31]	0.8 (0.4 – 1.4) [0.35]	1.1 (0.6 – 2.0) [0.89]
IV	0.5 (0.3 – 0.86) [0.009]	0.6 (0.4 – 1.0) [0.07]	0.8 (0.5 – 1.5) [0.53]
** Therapeutic modality**			
Surgery	*Ref.*	*Ref.*	*Ref.*
Radiation	0.8 (0.5 – 1.37) [0.33]	0.7 (0.4 – 1.0) [0.065]	0.7 (0.4 – 1.2) [0.2]
Chemoradiation therapy	0.6 (0.3 – 0.9) [0.025]	0.6 (0.5 – 1.0) [0.044]	0.6 (0.3 – 1.1) [0.075]

*Ref *Reference category

The univariate analyses show that patients with stage IV at diagnosis had a lower probability of RTW [SHR 0.5 (95% CI 0.3 – 0.9), *p* < 0.01] compared to patients with early stage cancer. Lastly, in comparison with surgery as unimodal therapy, receiving CRT in the 12 months after diagnosis was associated with a decreased probability of returning to work [SHR 0.6 (95% CI 0.3 – 0.9), *p* < 0.05].

The multicollinearity assessment (supporting aggregated data is made available in ‘Extra file 1’) showed that therapeutic modality and cancer stage were highly correlated. Sex was also correlated with household composition. Age was found to be highly and significantly correlated with all variables except for cancer stage and treatment modality and led to the decision to perform age-adjusted analyses for each of the explanatory variables separately.

The results of these analyses are presented in Table 2. After the inclusion of age in the univariate models, effects on the probability of RTW remained significant only for cohabitants with children compared to those without [SHR 1.9 (95% CI 1.1 – 3.6), *p* < 0.05] and CRT compared to surgery as unimodal therapy [SHR 0.6 (95% CI 0.5 – 1.0), *p* < 0.05].

The multivariate analysis, that included all explanatory variables simultaneously (Table 2), showed that only age and household composition were significantly associated with RTW. In comparison with HNC patients aged 36–50, being aged 18–25 or 26–35 is associated with a higher probability of RTW [SHR 3.3 (95% CI 1.9 – 5.8) and SHR 2.7 (95% CI 1.3 – 5.5) respectively, *p* < 0.001 and *p* < 0.01]. In the same vein, HNC patients aged 51 to 60 at diagnosis were significantly less likely to RTW over the follow-up compared to patients aged 36–50 [SHR 0.5 (95% CI 0.3 – 0.8), *p* < 0.05].

Furthermore, the mutually adjusted analyses demonstrated that cohabitating with child(ren) and living alone without a child increased the probability of RTW [respectively SHR 2.1 (95% CI 1.1 – 4.0) and SHR 2.2 (95% CI 1.1 – 4.5), *p* < 0.05] compared to cohabiting without children. The analyses showed that no other explanatory variables were significantly associated with the probability of RTW over follow-up. Considering the low number of individuals in the two youngest age categories, we grouped all individuals aged 18–35 and performed the univariate, age-adjusted and mutually adjusted analyses. These analyses did not produce significantly differing results, neither quantitatively nor qualitatively.

To further account for the effect of age on the RTW, multivariate analyses were stratified by redefined age categories (Table 3): (1) patients aged 50 years or younger at diagnosis and (2) patients older than 50 years. For both age groups, only the household composition remained significantly associated with RTW.

**Table 3:** Multivariate analyses of return to work after work inactivity following a head and neck cancer *diagnosis between 2004 and 2011* in Belgium, stratified by age group (≤ 50 years old and > 50 years old)

			**≤ 50 years old**			**> 50 years old**		
**Explanatory variables**			SHR	95% CI	*p*-value	SHR	95% CI	*p*-value
Sociodemographic explanatory variables	Sex	*ref. Man *	-	-	-	-	-	-
		Woman	1.6	0.9, 3.0	0.13	0.8	0.3, 2.2	0.66
	Household composition	*ref. Cohabitant*	-	-	-	-	-	-
		Cohabitant with child(ren)	1.2	0.5, 2.8	0.7	3.3	1.6, 6.8	0.002
		Single	0.8	0.3, 2.2	0.62	3.3	1.4, 7.8	0.008
		Single with child(ren)	0.8	0.3 2.3	0.62	0	0.0, 0.0	<0.001
Work-related explanatory variable	Occupational class	*ref. Blue collar*	-	-	-	-	-	-
		Civil servant	0.7	0.2, 2.2	0.49	0.5	0.1, 2.4	0.36
		Other	1.8	0.8, 4.0	0.14	1.8	0.7, 4.6	0.22
		White collar	1.4	0.5, 3.8	0.56	0.8	0.1, 4.2	0.75
Disease-related explanatory variables	Cancer stage	*ref. Stage l*	-	-	-	-	-	-
		Stage II	0.7	0.4, 1.5	0.42	0.6	0.2, 1.8	0.39
		Stage III	1.1	0.5, 2.4	0.78	1.4	0.5, 3.6	0.53
		Stage IV	0.7	0.3, 1.6	0.43	0.8	0.3, 1.9	0.64
	Treatment modality	*ref. Surgery*	-	-	-	-	-	-
		Radiation	1.3	0.7, 2.5	0.11	0.4	0.2, 1.1	0.064
		Chemoradiation	0.7	0.3, 1.7	0.44	0.6	0.3, 1.4	0.22

The number of events per variable (EPV) fell below the threshold of 40–50 put forth by Austin et al. [36].

## Discussion

In the present study, 21.61% of HNC patients who have known a period of work inactivity after their diagnosis became active on the labour market again during follow-up while 31.66% died, 6.53% got retired and 40.20% stayed inactive (censored). A systematic review and meta-analysis by Yu et al. [37] from 2022 on the RTW of HNC patients and their quality of life, found that 67% (95% CI: 62%—73%) of patients who were employed at diagnosis returned to work. Our study indicates a noticeably lower RTW among HNC patients. Moreover, none of the 21 included articles presented a RTW equal to or lower than ours. This RTW disparity could be explained by differences in patient and clinical characteristics (e.g., age, sex, stage at diagnosis, etc.) and study methods (e.g., highly differing follow-up times) but also by differences in countries’ incapacity schemes. Merely half of the studies in Yu et al.’s article applied an upper age limit and most did not take into account retirement as a competing event. Most notably though, all patients having undergone their treatment were eligible for inclusion while the present study included only those with a record of inactivity on the labour market. By applying this inclusion criterion however, the present study does not describe patients who remained active during the whole follow-up or until pension or death. After applying similar exclusion criteria for therapeutic modalities and missing values, this study finds that 1434 HNC patients remained active after a HNC diagnosis. Taking into account the individuals who RTW after a period of inactivity (*n* = 86), this means that 82.97% (1520 out of 1832 HNC patients) remained active or RTW over the follow-up. This should be taken into account when extrapolating insights from RTW rates that are described in this study. Importantly, when looking at the characteristics of the excluded population, this study finds that the distributions across categories of the explanatory variables are only significantly different to those for the included HNC population for therapeutic modality and occupational class.

The high number of excluded HNC patients results from the inability to take into account periods of inactivity shorter than one quarter due to the quarterly nature of the data. This means that the population for the current study design could more accurately be described as HNC patients who experience long-term inactivity (i.e. more than one quarter). Shorter periods of inactivity followed by RTW could not be identified and are assumed to have occurred within the HNC patients which were excluded. Finally, the current competing risk analyses do not allow for the assessment of repeated RTW or inactivity episodes. This would require the application of other types of analyses such as Multistate Life Tables and trajectory analyses [38].

In line with assumptions derived from the literature [24, 37], higher age was significantly and strongly associated with a lower RTW. The use of multivariate regression analyses within this study showed this association to be independent of all other included patient and clinical characteristics. One should take into account that, due to the relatively high median age of HNC patients (over 60 years old) in Belgium [4], younger age categories have a low number of patients and their results should be interpreted cautiously. The significant effect of sex on RTW probability in the univariate analysis was attenuated by the inclusion of age in the model, which is illustrated by the larger proportion of females in the younger age categories (16% of the female patients were under 36 years old compared to 2.81% of males).

Contrary to the majority of studies that investigated family circumstances [17], household composition was significantly associated with the RTW over the follow-up period. However, for HNC patients older than 50, cohabitating adults were much less likely to RTW compared to single patients and patients living with another adult and child(ren). An assumption could be that in the absence of children, cohabitating HNC patients can rely on the (financial) support of their cohabitant which is not possible for single patients. Similarly, the presence of a child can increase the financial burden on the family which could stimulate the RTW of HNC patients, which is suggested in literature for men but not for women [39]. However, this result was not reproduced when looking at patients aged 50 or less. Based on the findings of Austin et al., one should strive for a minimum of 40 to 50 primary events per variable (EPV) when modelling the effect of covariates of the cumulative incidence functions [36]. The current study did not meet this threshold. As such, the estimates produced by the Fine-Gray model in the current study cannot be assumed to be reliable. Still, due to the population-based nature of this study, increasing the sample sizes would be difficult, although less stringent exclusion criteria could potentially result in a larger sample.

Some literature suggests that blue collar workers are less likely to RTW compared to white collar workers [17, 37] possibly due to white collar working conditions and environments being more flexible and easier to adapt [40]. However, the present study found no significant effect of occupational class on the RTW probability in the univariate, age-adjusted nor multivariate analyses. Occupational class represents a widely used measure to assess socioeconomic differences in health outcomes [41, 42]. Although white vs. blue collar differences have previously been found for RTW probabilities in HNC patients [17], this study found no such effects of socioeconomic status when looking at occupational class. One should however, consider the unclear definition of the ‘other’ category of occupational class and its consequences on the validity of the current insights. Future studies should include detailed occupational class, socioeconomic status and educational level in their analyses to produce more reliable insights into RTW disparities.

Results from the present univariate analyses confirmed a diminished RTW probability, that can also be found in literature, associated with clinical factors such as advanced cancer stage and multimodality treatment (chemoradiation therapy) [17, 37]. The inclusion of age in the univariate models however, resulted in the attenuation of the effect of cancer stage while the multivariate analysis did the same for the effect of treatment modality. This suggests that, in the current study, clinical factors are not associated with the probability of RTW independently of age. We also found that the excluded HNC patients (i.e. those who remained active) showed significantly different distributions across treatment modalities compared to the included patients, where the excluded patients less often received multimodal treatment. This is surprising as a wide range of physical symptoms have been linked to RTW outcomes and because the anticipated symptom burden is, among others, determined by clinical cancer stage and its implications on therapeutic modalities [17]. Yet, most studies from the systematic review of Morales et al. that included clinical factors, found similar insignificant results [17]. However, it is important to note that our study did not have direct information on symptom burden. Future studies would benefit the direct assessment of (physical) symptoms to clarify its impact on RTW. Importantly, one also needs to consider that more detailed descriptions of clinical factors, and especially surgery, are required for more accurate and valid results. Due to the inability to directly define surgery, which was thus based on assumptions, the current study has no measure for its application in patients who received chemotherapy or radiation therapy. In reality, 18.9% of Belgian HNC patients received surgery only compared to 24.87% in the current study [4]. Either as unimodal or part of a multimodal treatment regimen, 38.1% of Belgian HNC patients underwent surgery between 2009 and 2014 [4].

### Strengths and limitations

The data was representative and originated from population-based registries. This study used a strong retrospective longitudinal cohort design contrary to most studies in the field with a maximum follow-up period of 7 years and 9 months and average follow-up time of 7 quarters after diagnosis. The use of multicollinearity checks, age-adjusted regression models, (stratified) multivariate regression models and the inclusion of competing events in our analyses represent important strengths. However, several significant limitations exist. The inability to directly define surgery limits the extrapolation of real-life insights as we cannot distinguish between multimodal treatments that include or exclude surgery. Also, our dataset does not include information on therapies that took place outside Belgium. The impact of such therapies cannot be estimated as information on the proportion of patients receiving therapies outside of Belgium is (not publicly) available. Having deducted and assumed the retirement status (as a competing event) represents another limitation, although its calculation is in line with current trends of inactivity on the labour market among those older than 50 in Belgium [29]. Furthermore, the quarterly nature of the data does not allow for the identification of labour market transitions that took less than one quarter, i.e. the present result address long-term inactivity. The number of patients having ‘other’ as occupational class deserves more attention and clarification in order to produce more accurate results. A large proportion of the sample was excluded because of missing values for cancer stage, further reducing an already limited study population. Although our study described the most complete eligible HNC patient population possible in Belgium, the number of EPV did not meet the requirements described by Austin [36] as a result of too few cases of RTW. Future studies might consider a longer follow-up period and less stringent exclusion criteria to attain larger sample sizes.

## Conclusion

The RTW following a HNC diagnosis after a period of inactivity was 21.61%, which is lower than RTW probabilities that can be found in literature for both HNC patients and the cancer patients in general. Although excluded from the study population, significantly more patients remained active throughout the follow-up than those having RTW after a period of inactivity.

Multivariate competing risk regression analyses (Fine-Gray proportional hazards models) showed only age and household composition to be independently associated with RTW probability. Furthermore, the stratified analysis showed that the effects of household composition are different for those aged 50 or less and those older than 50, although not significant for those of 50 years and younger. Importantly, no association was found between RTW probability and both work-related and clinical factors (stage and treatment modalities). Future studies covering this topic would benefit from a similar study design, but more detailed measures of therapeutic modalities, job-related characteristics and socioeconomic status. Our study highlights the need for more policy efforts to support the RTW of HNC patients who, following their diagnosis, have been inactive on the labour market for more than one quarter. As age and household composition represent factors that are not modifiable, policies or interventions that address the RTW of HNC patients should be tailored to support specific target groups according to the socio-demographic profiles of the patients.

## Supplementary Information

Supplementary Material 1.

## Data Availability

The raw data that support the findings of this study are not openly available due to reasons of sensitivity. Aggregated data can be found ‘Extra file 1’ in the form of spreadsheets (.xls file).
